# Assessment of Factors Regulating Androgen Receptor (AR) and Estrogen Receptor Alpha (ERα) Expression in Men with End-Stage Hip Osteoarthritis

**DOI:** 10.3390/ijms27125294

**Published:** 2026-06-11

**Authors:** Aleksandra Rył, Marta Grabowska, Alina Jurewicz, Andrzej Bohatyrewicz, Aleksandra Szylińska, Weronika Ratajczak, Małgorzata Piasecka, Katarzyna Michałek, Anna Lubkowska, Iwona Rotter

**Affiliations:** 1 Department of Medical Rehabilitation and Clinical Physiotherapy, Pomeranian Medical University in Szczecin, 54 Żołnierska, 71-210 Szczecin, Poland; iwona.rotter@pum.edu.pl; 2 Department of Histology and Developmental Biology, Pomeranian Medical University in Szczecin, 48 Żołnierska, 71-210 Szczecin, Poland; marta.grabowska@pum.edu.pl (M.G.);; 3 Department of Specialized Nursing, Pomeranian Medical University in Szczecin, 48 Żołnierska Street, 71-210 Szczecin, Poland; 4 Department of Orthopedics, Traumatology and Musculoskeletal Oncology, Pomeranian Medical University in Szczecin, 1 Unii Lubelskiej, 71-252 Szczecin, Poland; 5 Department of Cardiac Surgery, Pomeranian Medical University in Szczecin, 72 Powstańców Wielkopolskich, 70-111 Szczecin, Poland; 6 Department and Division of Functional Diagnostics and Physical Medicine, Pomeranian Medical University in Szczecin, 54 Żołnierska, 71-210 Szczecin, Poland; 7 Department of Physiology, Cytobiology and Proteomics, Faculty of Biotechnology and Animal Sciences, West Pomeranian University of Technology in Szczecin, 29 Klemensa Janickiego, 71-270 Szczecin, Poland

**Keywords:** androgen receptor (AR), estrogen receptor alpha (ERα), bone tissue, osteoarthritis (OA)

## Abstract

This study aimed to identify environmental and hormonal determinants of steroid receptor expression in the bone tissue of men with osteoarthritis. Twenty-six men undergoing total hip arthroplasty were included. Serum hormones, bone turnover markers, and elemental concentrations were assessed, and bone samples were analyzed using immunohistochemistry and multivariate regression models. Results: Estrogen receptor alpha (ERα) expression was a significant positive predictor of the bone formation marker procollagen type I N-terminal propeptide (PINP) (*p* = 0.0386), indicating its key role in anabolic processes. Androgen receptor (AR) expression showed a positive trend with the resorption marker CTX-I (*p* = 0.0956). A strong negative association was observed between Body Mass Index (BMI) and ERα expression (β = −0.50; *p* = 0.0018), suggesting reduced estrogen sensitivity in individuals with higher adiposity. Local accumulation of metals significantly influenced receptor expression. Lead in bone was strongly associated with increased AR expression (β = +1.457; *p* = 0.0009), whereas manganese was positively associated with ERα expression (β = +2.112; *p* = 0.0409). These findings indicate that local bone tissue analysis provides more accurate insight into hormonal regulation than serum measurements and highlight the role of environmental factors in modulating bone metabolism in men.

## 1. Introduction

The maintenance of bone tissue homeostasis in men is a multifactorial process in which the regulation of steroid receptor-dependent signaling pathways—androgen receptor (AR) and estrogen receptors (ERα and ERβ)—plays a central role. Although testosterone (T) is the principal circulating androgen, evidence from both genetic models and clinical studies of men with aromatase deficiency or ER mutations indicates that estrogens, generated through peripheral aromatization of testosterone, play a dominant role in regulating bone metabolism and maintaining bone mass [1]. Estrogens (E2) are critical for inhibiting bone resorption, particularly in cortical bone [2]. In contrast, androgens acting directly via AR are essential for maintaining trabecular bone structure and promoting periosteal apposition. Receptors for both sex hormones have been identified in all bone cell types, with AR predominantly expressed in osteoblasts and osteocytes, where it regulates proliferation, differentiation, and extracellular matrix synthesis, including type I collagen and osteocalcin [3].

The genetic basis of nuclear receptor expression constitutes a fundamental layer of susceptibility in joint tissue degeneration. Variations in genes encoding the androgen receptor (AR) and estrogen receptors can directly correlate with systemic parameters of skeletal health and susceptibility to bone mass loss, as highlighted by clinical investigations mapping exon configurations and polymorphic variations in these hormone-sensing genes [4]. Furthermore, the wider application of nutrigenomics and precision screening tools demonstrates that genetic polymorphisms significantly dictate individual cellular responses, inflammatory pathways, and oxidative stress outcomes within musculoskeletal networks during osteoarthritis progression [5].

Estrogens play a fundamental role in maintaining skeletal integrity and joint homeostasis by regulating the balance between bone formation and resorption. At the cellular level, estrogen signaling promotes osteoblast survival, inhibits osteocyte apoptosis, and suppresses osteoclastogenesis via the down-regulation of RANKL. While estrogens support the anabolic microenvironment required for osteoblast differentiation and the subsequent synthesis of extracellular matrix, the systemic level of PINP (N-terminal propeptide of type I procollagen) serves as a sensitive marker of total bone turnover and osteoblastic anabolic rate, reflecting the cleavage of procollagen during bone matrix deposition rather than a direct, isolated product of estrogen action alone. Concurrently, androgen signaling exhibits a complex, dual nature in bone tissue and joint biology. Androgens exert direct anabolic effects on the skeleton by stimulating osteoblast proliferation and differentiation through the androgen receptor (AR). However, their role in osteoarthritis (OA) and cartilage maintenance appears multi-faceted and contradictory. While testosterone is essential for muscle mass and joint loading distribution, high genetically predicted levels of sex steroids, particularly testosterone, have been paradoxically linked to an increased risk of developing hip and knee osteoarthritis in men, as demonstrated by recent Mendelian randomization studies [6]. This dual nature suggests that while optimal androgen signaling supports bone density, its systemic and local variations may differentially modulate cartilage degradation and subchondral bone remodeling in a joint-specific manner.

Beyond genomic predispositions, bone tissue is an acutely mechanosensitive structure where physical forces directly modulate the density and stability of steroid pathways. Standard physiological loading and training-induced interventions act through intricate cellular networks to regulate osteogenic differentiation and maintain bone mass through the specialized mediation of non-coding RNAs [7]. Mechanistic laboratory studies have verified that fluid shear stress and localized mechanical strain exert a strong synergistic impact with circulating hormones, demonstrating that mechanical forces directly augment intracellular cascades, signal transduction, and the transcriptomic expression of key osteoblast markers via estrogen receptor-mediated pathways [8]. Consequently, standard or altered weight-bearing dynamics across the knee and hip joints dictate localized mechanotransduction, linking mechanical rehabilitation directly to the molecular responses of subchondral bone cells [9].

Bone tissue also serves as a major reservoir for minerals and metals that can modulate local signaling pathways. Among these, manganese (Mn) is essential for bone matrix synthesis and for protecting cells against oxidative stress via manganese superoxide dismutase (MnSOD) [6]. Iron homeostasis is likewise critical, as excess iron promotes the generation of reactive oxygen species, thereby enhancing bone resorption while inhibiting bone formation [10].

Crucially, the local expression profiles of steroid receptors and subsequent tissue responsiveness to hormonal signals do not operate in isolation; they are continuously modified by the inflammatory and transcriptional microenvironment. In the chronic state of osteoarthritis, the joint transforms into a multi-faceted network governed by pathological signaling pathways, including localized elevations of pro-inflammatory cytokines, which drive tissue dysfunction across all joint components [11]. These inflammatory markers directly cross-talk with nuclear proteins, a process intricately balanced by specific structural components such as steroid receptor coactivator-1 (SRC-1) and the p160 family of coactivators, which are essential for integrating steroid signals, enhancing transactivation, and preventing receptor degradation [12]. Alterations in these coactivator networks significantly influence long-term cellular responses and tissue integrity during chronic degenerative pathologies [13]. Furthermore, the direct clinical interplay between osteoarthritis and systemic bone remodeling disorders underscores the concept that chronic joint stress, subchondral sclerosis, and shifting mechanical vectors modify local tissue sensitivity independently of baseline circulating hormone volumes [14].

The novelty of this study lies in its focus on steroid receptor expression assessed directly in human bone tissue obtained intraoperatively, enabling the evaluation of local metabolic processes not captured by standard serum-based analyses. The originality of this work further derives from the integration of immunohistochemical assessment with in situ analysis of heavy metal and trace element accumulation, addressing an important gap in understanding factors that modulate the hormonal sensitivity of bone tissue in men. The primary aim was to comprehensively evaluate AR and ERα expression in the bone tissue of men undergoing total hip arthroplasty. The study also sought to identify determinants of local receptor density, with particular emphasis on heavy metal accumulation and its relationship with bone turnover markers.

## 2. Results

### 2.1. General Characteristics of the Study Group and Biochemical Profiles

Table 1 presents the anthropometric characteristics as well as the biochemical profiles of the study group of men, expressed as medians and interquartile ranges.

Regarding the clinical reference ranges of the assessed serum parameters, the median values for TT and E2 remained within standard physiological limits for aging men. However, a significant subset of patients exhibited elevated bone turnover markers. Specifically, 38% (*n* = 10) of the participants presented with serum PINP levels exceeding the upper limit of the standard reference range, while 15% (*n* = 4) showed elevated CTX-I levels, reflecting the active subchondral bone remodeling characteristic of end-stage osteoarthritis.

A univariate screening using Spearman’s rank correlation coefficients (rs) was performed to evaluate the direct relationships between tissue steroid receptors, local element accumulation, and metabolic parameters. The correlation coefficients and their corresponding significance levels are summarized in Table 2. Local ER**α** expression in bone tissue demonstrated a significant positive correlation with systemic PINP levels (rs = 0.279, *p* = 0.038), confirming a link with systemic bone formation markers. Concurrently, a strong, highly significant positive correlation was identified between local AR tissue density and intracellular lead accumulation (rs = 0.415, *p* = 0.001), supporting the interplay between heavy metal deposition and receptor presentation. In contrast, local ER**α** density exhibited a pronounced negative correlation with BMI (rs = −0.442, *p* = 0.002), indicating that increased adiposity is linked to a downregulation of estrogenic pathways within the joint microenvironment. A statistically significant positive correlation was also verified between local ER**α** expression and tissue manganese deposition (rs = +0.312, *p* = 0.022).

### 2.2. Immunohistochemical Analysis of AR and ERα Expression

**Figure 1:**
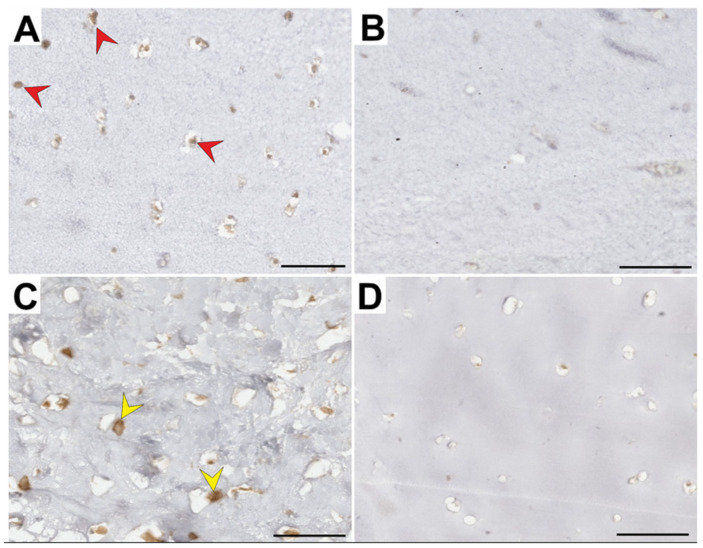
Immunoexpression of steroid hormone receptors in human bone tissue. Representative microphotographs showing immunoexpression of (**A**) androgen receptor (AR) and (**C**) estrogen receptor alpha (ERα) in trabecular bone. Brown-stained nuclei indicate positive AR (red arrowheads) and ERα (yellow arrowheads) immunoexpression in bone cells. (**B**,**D**) Negative controls. Scale bar—50 µm.

The detailed immunohistochemical expression profiles, including the minimal, maximal, and median positive cell percentages for both AR and ERα, are summarized in Table 3, while their spatial and cellular distribution are illustrated in Figure 1. The percentage of cells showing positive immunoreactivity for androgen receptors (AR) was noticeably higher, with a median density of 10.140%, compared to estrogen receptors alpha (ERα), which exhibited a median positive cell count of 3.473%, while chronological age and BMI are detailed as baseline characteristics elsewhere.

### 2.3. Multivariate Predictors of AR Expression in Bone Tissue

To identify determinants of local receptor status in bone tissue, backward stepwise regression analyses were performed for AR and ERα. Initial models for both receptors included the same set of variables: age, anthropometric indices (BMI, WHR), and the complete serum hormonal profile (total and free testosterone, estradiol, SHBG, and DHEA).

In both models, total testosterone (TT), free testosterone (FT), and waist-to-hip ratio (WHR) were excluded from the final equations, indicating the absence of a direct association between systemic sex hormone levels and local receptor expression in the bone matrix of the studied men. Despite this similarity, the final models revealed distinct regulatory patterns for the two receptors. For AR (Table 4), age emerged as the principal determinant, showing a positive trend (β = +0.53; *p* = 0.055). This model was additionally characterized by a marginal trend for DHEA (negative, *p* = 0.076), whereas estradiol showed no statistically significant association (*p* = 0.094) and BMI was excluded from the final equation.

In contrast, a different set of predictors was identified for the estrogen receptor (ER) (Table 4). In this model, age was not significant, while BMI was the dominant and highly significant negative predictor (β = −0.50; *p* = 0.0018). Additional significant negative predictors of ER expression included SHBG (β = −0.03; *p* = 0.0233) and DHEA (β = −1.74; *p* = 0.0402). Serum estradiol concentration was the only variable positively associated with ER expression (β = +0.03; *p* = 0.0097).

### 2.4. Multivariate Predictors of ERα Expression in Bone Tissue

To assess the relationship between local receptor status and bone metabolic activity, backward stepwise regression analyses (Table 5) were performed for markers of bone formation (PINP) and resorption (CTX-I). Initial models included AR and ER expression, along with control variables (age and BMI), to identify the strongest determinants of bone turnover.

In the model for the bone formation marker (PINP), age, BMI, and AR expression were excluded as non-significant. ERα remained the only independent and statistically significant positive predictor of PINP levels (*p* = 0.0386).

For the bone resorption marker (CTX-I), age, BMI, and ERα expression were also excluded, as none showed an association with bone matrix degradation. The final model retained only AR expression, which exhibited a positive trend (β = +0.007; *p* = 0.0956).

To identify determinants of steroid receptor density in the studied men, multistep backward stepwise regression analyses were performed. Both serum-derived parameters and local (bone tissue) variables were included, and all models were adjusted for age and BMI (Table 6).

In the serum model for AR, systemic mineral homeostasis emerged as a significant regulatory factor. Phosphorus (P) showed a significant positive association (β = 0.08; *p* = 0.0093), whereas magnesium (Mg) was negatively associated (β = −0.92; *p* = 0.0283). No statistically significant association was observed for iron (Fe) (*p* = 0.1287), and other macroelements (Ca, Na, K, Zn, Cr) were excluded from the model as non-significant.

At the tissue level, the model demonstrated markedly higher explanatory power (adjusted R^2^ = 0.88), indicating a stronger contribution of local factors. Accumulation of metals within bone tissue emerged as the primary determinant. Potassium (K) (β = +0.013; *p* = 0.0001) and lead (Pb) (β = +1.457; *p* = 0.0009) were identified as the strongest positive predictors. The latter finding is of particular clinical relevance, suggesting a robust association between lead accumulation and increased AR expression. Age remained a significant positive predictor in both serum and bone models (β = +0.760; *p* = 0.0013 in the bone model), whereas BMI was excluded as non-significant.

In contrast, ERα expression exhibited a distinct predictive pattern. Within the serum model, no statistically significant mineral predictors were identified, with no statistically significant associations observed for copper (Cu) (*p* = 0.1037) or BMI (*p* = 0.1178).

In the bone tissue model (adjusted R^2^ = 0.22), manganese (Mn) was the only variable retained as a significant predictor, showing a positive association with ERα expression (β = +2.112; *p* = 0.0409). In contrast to AR, age (*p* = 0.2991), BMI (*p* = 0.8698), and lead were excluded as non-significant predictors.

## 3. Discussion

The primary aim of this study was to identify determinants of local steroid receptor density in the bone tissue of men, thereby providing insight into the pathomechanisms of degenerative changes from an environmental toxicology perspective. The findings demonstrate that peripheral blood parameters do not reflect the actual exposure of bone cells to heavy metals, which may account for inconsistencies in previous epidemiological studies based predominantly on urine or serum analyses [15]. Only direct assessment within the bone matrix revealed strong and statistically significant associations, indicating that local deposition of these elements is a key modulator of hormonal sensitivity and that their long-term accumulation may directly alter bone cell phenotype and regenerative capacity [16].

In the analyzed tissue, a statistically significant positive association was observed between ERα expression and the concentration of the N-terminal propeptide of type I procollagen (PINP) (*p* = 0.0386). This finding, derived from multivariate regression analysis, identifies local ERα density as an independent and principal determinant of bone formation potential in men, exceeding the influence of chronological age and BMI. Analysis of factors determining receptor density also revealed a strong association with body mass. A pronounced and statistically significant negative effect of BMI on ERα expression was identified (*p* = 0.0018; β = −0.50), representing one of the key findings of this study. This suggests a paradoxical mechanism in men: although obesity may increase the availability of estrogens through peripheral aromatization in adipose tissue, it simultaneously reduces bone tissue sensitivity to these hormones by decreasing receptor density.

These findings indicate that under conditions of excessive adipose tissue accumulation (high BMI), this protective mechanism is disrupted at the receptor level. Despite potentially increased ligand availability (estradiol), receptor availability in bone is reduced, limiting the tissue’s responsiveness to pro-regenerative hormonal signaling. This process may be driven by chronic inflammation and oxidative stress associated with obesity, which have been shown to impair steroid hormone signaling pathways and adversely affect bone homeostasis [17,18]. Consequently, elevated BMI in men, rather than conferring protection, may paradoxically contribute to bone degeneration through reduced functional responsiveness to estrogens.

This receptor downregulation is further compounded by the inflammatory microenvironment characteristic of chronic joint disease. Advanced osteoarthritis establishes a complex network of pathological signaling cascades, where localized elevations of pro-inflammatory cytokines compromise cellular function across all joint components [11]. These inflammatory mechanisms actively cross-talk with nuclear steroid receptors, a transcriptomic process heavily modulated by coactivator pathways—such as steroid receptor coactivator-1 (SRC-1) and the p160 family—which integrate hormonal signals and regulate receptor transactivation under persistent stress [12,13].

As bone tissue accumulates environmental toxins, their presence may directly influence steroid receptor density and local hormonal responsiveness. One of the key findings of this study is the identification of a strong link between environmental toxicology and the endocrine regulation of bone tissue. Multivariate regression analysis revealed a statistically significant positive association between lead (Pb) accumulation in bone and AR expression (β = +1.457; *p* = 0.0009), with the model incorporating metals (Pb, K) explaining up to 88% of the variability in receptor expression. This finding indicates that lead does not act solely as a cytotoxic agent but also exhibits endocrine-disrupting properties, selectively modulating signaling pathways in bone tissue. Second, as an endocrine-disrupting compound, lead may interact directly with receptor domains or affect receptor stability, thereby triggering compensatory upregulation of receptor synthesis in response to impaired signaling [18].

Our findings suggest that lead-induced AR overexpression reflects a pathological mechanism resembling tissue-level androgen resistance. Lead accumulation may disrupt functional AR signaling (e.g., by impairing DNA binding or coactivator interactions), prompting compensatory receptor overproduction. This excess of dysfunctional or improperly activated receptors may, rather than inhibiting resorption, paradoxically promote bone matrix degradation and accelerate catabolic remodeling, as supported by the observed association with CTX-I.

Concurrently, this element-driven endocrine disruption occurs alongside altered biomechanical stresses acting upon the subchondral bone and joint architecture. Physical forces and physiological loading act as essential regulators of bone modeling, driving osteogenic differentiation via intracellular networks and specific non-coding RNAs [7,9]. Biomechanical strain and fluid shear stress have been verified to expand intracellular cascades and target gene expression, operating synergistically through estrogen receptor-dependent pathways and integrin-mediated mechanotransduction [8].

In contrast to the toxic effects of lead, manganese (Mn) accumulation in bone tissue appears to exert a protective influence on the local hormonal environment. In multivariate analysis, manganese was the only element in the bone matrix that remained a significant positive predictor of ERα density (β = +2.112; *p* = 0.0409). Our findings indicate that local manganese levels in bone tissue act as a significant positive predictor of ERα expression. This relationship suggests that manganese may play a supportive role in modulating the local density or stability of estrogen receptors within the arthritic bone microenvironment, thereby potentially altering tissue responsiveness to estrogenic signaling. This observation aligns with a recent comprehensive meta-analysis by Shi et al., which demonstrated that manganese concentrations are significantly depleted in patients with osteoarthritis compared to healthy controls [19]. While Shi et al. highlighted a systemic manganese deficiency as a potential risk factor or feature of OA progression, our data expand on this by showing that even within an arthritic cohort, intra-tissue variations of manganese are tightly linked to the molecular regulation of sex hormone receptors. Mechanistically, this effect may be attributed to the role of manganese as a cofactor of antioxidant enzymes, particularly manganese-dependent superoxide dismutase (MnSOD) localized in the mitochondria of bone cells. MnSOD protects osteoblasts and osteocytes from oxidative stress by neutralizing reactive oxygen species (ROS), which inhibit osteoblast differentiation and promote degradation of receptor proteins. In addition, manganese activates integrins, enhancing osteoblast adhesion and survival, which may contribute to the stabilization of nuclear receptors and preservation of the anabolic potential of bone tissue [16]. These findings support the view that physiological manganese deposition in bone serves a cytoprotective function, preserving estrogen signaling pathways from degeneration.

The analysis also revealed a link between systemic mineral metabolism and local AR density. AR expression in bone was positively associated with serum phosphorus (P) levels (*p* = 0.0093), whereas magnesium (Mg) was identified as a significant negative predictor (*p* = 0.0283).

The results further indicate that steroid receptors in bone tissue perform two distinct yet complementary roles. ERα appears to be the primary driver of anabolic processes and new tissue formation. This is supported by the finding that local ERα density was the only independent and statistically significant predictor of PINP levels (*p* = 0.0386), positioning the estrogen signaling pathway as the principal regulator of collagen synthesis in men. In contrast, AR in the studied group showed a stronger association with bone turnover dynamics and resorption. In the regression model for CTX-I, AR remained a predictor with a positive trend (*p* = 0.0956), while age and BMI were excluded.

Despite the high coefficients of determination observed in the models (e.g., adjusted R^2^ = 0.88 for AR), several limitations should be acknowledged. The sample size was relatively small (*n* = 26), reflecting the unique nature of the biological material. The study was exploratory, and the limited cohort may have reduced statistical power to confirm some observed trends. Moreover, the ex vivo design, combined with the complexity of the biochemical and immunohistochemical analyses, may limit direct extrapolation of the findings to systemic physiology. Finally, the study population was relatively homogeneous, with a median age of 66 years and median BMI of 29.2, and thus the findings (e.g., the strong effect of age on AR expression) should be interpreted within the context of this specific cohort.

Consequently, the elemental and anthropometric variations identified in our regression models operate on a background of pre-existing age-related skeletal changes. This background is strongly dictated by the clinical staging and long-term chronicity of osteoarthritis, where chronic joint stress and subchondral remodeling modify local tissue sensitivity independently of baseline circulating hormone volumes [14]. Furthermore, this timeline intersects with individual genetic susceptibility, as specific polymorphic variations and microsatellite polymorphisms in genes encoding AR and ER dynamically correlate with baseline bone mineral density and modulate distinct inflammatory or oxidative stress responses during degeneration [4,5].

Additionally, several limitations of this study must be acknowledged. First, baseline systemic Bone Mineral Density (BMD) measurements were not available for this patient cohort, which precludes the correlation of local molecular receptor expression with macroscopic bone density parameters. Second, our study lacks a healthy control group. Obtaining healthy, non-arthritic bone tissue from the proximal femur of age-matched men presents major ethical and technical challenges, as bone sampling is restricted to surgical waste collected during joint replacement. Consequently, our findings are highly specific to patients with advanced osteoarthritis and cannot be directly extrapolated to a healthy population. Future multi-center studies incorporating standardized pre-operative osteodensitometry and alternative control tissues are warranted to further expand on these pathways.

## 4. Materials and Methods

### 4.1. Study Group Characteristics

The study comprised 26 men scheduled for total hip arthroplasty due to advanced osteoarthritic changes at the Department of Orthopedics, Traumatology and Musculoskeletal Oncology, Pomeranian Medical University in Szczecin. To ensure methodological rigor, the following exclusion criteria were applied: diabetes mellitus, a history of malignancy, alcohol abuse, hepatic or renal insufficiency, and heart failure classified as New York Heart Association (NYHA) class III or IV. Patients receiving medications known to affect bone metabolism were also excluded, including mineral supplements, neuroleptics, cytostatic agents, immunosuppressive drugs, corticosteroids, and antidepressants. The median age of the participants was 66.0 years (IQR: 64.0–71.0 years). The group was characterized by a median body mass index (BMI) of 29.2 kg/m^2^ (IQR: 27.1–31.0 kg/m^2^).

### 4.2. Study Material

#### Collection and Preparation of Biological Material

Venous blood samples were collected in the morning on the day of surgery following an overnight fast. Blood was drawn into sterile tubes and centrifuged to separate serum. The serum samples were then frozen and stored at −80 °C until analysis of hormonal parameters, bone turnover markers, and elemental concentrations.

Bone tissue samples, consisting of femoral head fragments, were obtained intraoperatively during total hip arthroplasty. The collected tissue was subdivided according to the requirements of the planned analyses.

### 4.3. Determination of Hormone Concentrations and Bone Turnover Markers in Blood

Venous blood samples were collected from all participants after an overnight fast (between 07:00 and 09:00) via standard venipuncture into clot-activator tubes. Following collection, the samples were allowed to clot at room temperature for 30 min and then centrifuged at 3000 rpm for 10 min at 4 °C. The separated serum was immediately aliquoted and stored at −20 °C until analysis. Concentrations of total testosterone (TT; reference range for males: 2.36–9.96 ng/mL), estradiol (E2; 11.2–50.4 pg/mL), sex hormone-binding globulin (SHBG; 18–110 nmol/L), and dehydroepiandrosterone sulfate (DHEAS; 110–470 μg/dL) were determined using commercially available ELISA assays (DRG Medtek, Warsaw, Poland) strictly according to the manufacturer’s instructions. Free testosterone (FT; 8.9–45.5 pg/mL) was calculated according to the formula proposed by Vermeulen: FT = (TT − N − SHBG + √((N + SHBG − TT)^2^ + 4NT))/2N, where N = 0.5217 × albumin concentration + 1 [20]. Bioavailable testosterone (bioT) was calculated using the method described by Morris et al. [21]. The following bone turnover markers were measured in serum: osteocalcin (OC; 5–25 ng/mL), parathyroid hormone (PTH; 10–60 pg/mL), C-terminal telopeptide of type I collagen (CTX-I; 0.115–0.748 ng/mL), and procollagen type I N-terminal propeptide (PINP; 85.55–2028.75 ng/mL) using dedicated, highly specific solid-phase ELISA test kits from the same manufacturer. All immunoassays were processed uniformly, and the optical densities were quantified using an automated microplate spectrophotometric reader at a wavelength of 450 nm, with a reference wavelength correction at 620 nm (DRG Medtek, Warsaw, Poland). To ensure analytical reliability and reproducibility, all standards, controls, and patient serum samples were assayed in duplicate. The internal quality validation demonstrated acceptable assay precision, with the intra-assay and inter-assay coefficients of variation (CV%) consistently remaining within acceptable thresholds of less than 8.5% and 10.0%, respectively.

### 4.4. Elemental Analysis in Bone Tissue and Serum

Serum and bone samples were stored at −80 °C until analysis. Concentrations of elements in serum (calcium—Ca, magnesium—Mg, and phosphorus—P) and in bone tissue (lead—Pb, cadmium—Cd, manganese—Mn, zinc—Zn, copper—Cu, iron—Fe, and potassium—K) were determined using inductively coupled plasma optical emission spectrometry (ICP-OES; iCAP™ 7400, Thermo Fisher Scientific, Waltham, MA, USA). This method is widely recognized for the quantitative analysis of trace elements in both liquid and solid matrices. Measurements were performed in both radial and axial modes.

Serum samples were thawed at room temperature and digested using a CEM MARS 5 microwave digestion system (CEM Corporation, Matthews, NC, USA). Bone samples were thawed at room temperature, then dried at 80 °C overnight to constant weight after removal of adhering soft tissues. The dried samples were ground to a fine powder using a porcelain mortar and subjected to mineralization using the CEM MARS 5 system. A minimum of 0.1 g of bone tissue was pre-reacted for 30 min under a fume hood, followed by the addition of 1 mL of unstabilized 30% H_2_O_2_. Samples were placed in Teflon vessels (CEM Corporation, Matthews, NC, USA) and digested in a microwave system at 180 °C for 35 min. After digestion, samples were cooled to room temperature.

Blank samples were prepared by adding concentrated nitric acid to empty tubes and processed identically to the study samples. Multi-element calibration standards (ICP multi-element standard solution IV, Merck, Darmstadt, Germany) were prepared in the same manner as blanks and samples. Deionized water (Direct-Q UV, Merck, Darmstadt, Germany; resistivity approximately 18.0 MΩ·cm) was used for all solution preparation. A certified reference material consisting of steam-treated bone meal (Merck, Darmstadt, Germany), sieved and homogenized, was used for calibration of bone samples. Quality control for serum trace element analysis was performed using SRM 8414 (NIST Bovine Muscle, Gaithersburg, MD, USA), analyzed by instrumental neutron activation analysis.

### 4.5. Immunohistochemical (IHC) Reaction

Bone samples were fixed in 10% neutral-buffered formalin for at least 24 h at room temperature. To allow thin sectioning while preserving tissue architecture and antigenicity for nuclear steroid receptors, the fixed bone fragments were subsequently subjected to chemical decalcification using a stabilized aqueous solution containing 10% acetic acid and formalin at room temperature. The decalcification fluid was refreshed every 3–4 days over a period of approximately 2–3 weeks until a complete loss of tissue mineral density was verified via physical elasticity testing. Following decalcification, the specimens were thoroughly rinsed in running tap water, dehydrated through a graded ethanol series, cleared in xylene, and embedded in paraffin blocks. Serial sections (3–5 μm) were cut using a microtome (Microm HM340E, Thermo Fisher Scientific, Waltham, MA, USA) and mounted on poly-L-lysine–coated microscope slides (Thermo Scientific, Loughborough, UK; cat. no. J2800AMNZ).

For antigen retrieval, deparaffinized and rehydrated sections were heated in a water bath for 30 min in Target Retrieval Solution (Dako Cytomation, Carpinteria, CA, USA; cat. no. S2367). After cooling and rinsing in PBS, endogenous peroxidase activity was blocked using Peroxidase Blocking Solution (Dako Cytomation, Carpinteria, CA, USA) for 10 min. Sections were then incubated for 30 min in a humidified chamber with primary antibodies against AR (Dako Cytomation, clone AR441, M3562; dilution 1:50) and ERα (Dako Cytomation, Carpinteria, CA, USA, Cytomation, clone EP1, M3643; dilution 1:40). Antibodies were diluted in a background-reducing antibody diluent (Dako Cytomation, Carpinteria, CA, USA, cat. no. S3022).

Subsequently, sections were incubated with horseradish peroxidase–conjugated secondary antibodies (Dako REAL™ EnVision™ Detection System Peroxidase/DAB+, Rabbit/Mouse). Immunoreactivity was visualized using diaminobenzidine (DAB). Sections were rinsed in distilled water and counterstained with hematoxylin. Negative controls were processed identically, omitting the primary antibodies. Positive staining was evaluated microscopically (BX41, Olympus Optical, Tokyo, Japan) based on the presence of a brown chromogenic signal.

### 4.6. Quantitative Computer-Assisted Image Analysis of Histological Slides

Following IHC staining, slides were scanned using an Ocus slide scanner (Grundium, Germany) at 800× magnification (0.25 μm/pixel resolution). Digital images were analyzed using ImageScope software (version 11.2.0.780; Aperio Technologies, Vista, CA, USA). Quantification of AR-positive and ERα-positive cells was performed using the Nuclear Algorithm v9 (version 9.1; Aperio Technologies). Algorithm parameters, including intensity thresholds for positive staining, were adjusted to align with visual assessment. Regions of interest were defined manually. The percentages of AR-positive and ERα-positive cells were calculated independently across 2–5 fields per patient (61 fields in total), with mean analyzed areas of 0.57 mm^2^ (AR) and 0.64 mm^2^ (ERα).

### 4.7. Statistical Analysis

Statistical analyses were conducted using Statistica software (version 13.3, StatSoft, Poland). Normality of distribution was assessed using the Shapiro–Wilk test. As most variables deviated from normal distribution, descriptive data are presented as medians with interquartile ranges (IQR: Q1–Q3). Determinants of steroid receptor expression in bone tissue were evaluated using multivariate modeling based on backward stepwise regression with bootstrap resampling (1000 iterations). This approach enabled robust estimation of parameter significance despite non-normal data distribution. A *p*-value < 0.05 was considered statistically significant, while values in the range 0.05 < *p* < 0.10 were interpreted as statistical trends.

## 5. Conclusions

In conclusion, this study demonstrates that the local expression of steroid receptors in the bone tissue of men with advanced osteoarthritis is significantly determined by a complex interplay of environmental, elemental, and metabolic factors, operating independently of systemic hormone concentrations. Multivariate modeling revealed that elevated lead (Pb) and potassium (K) content in bone tissue serve as the strongest predictors for increased AR expression. Conversely, higher local levels of manganese (Mn) combined with a lower Body Mass Index (BMI) significantly predict higher estrogen receptor (ERα) expression. These findings highlight the multi-faceted nature of the bone microenvironment in osteoarthritis, where toxic and essential elements interact with cellular signaling. This underscores the need to look beyond systemic endocrine profiles when investigating joint pathology and bone remodeling in aging men.

## Figures and Tables

**Table 1:** Anthropometric characteristics and profiles of hormonal parameters, bone turnover markers, and element concentrations in serum and bone tissue in the study group of men.

Variable		*N* = 26		
		Me	Q1	Q3
Serum hormone concentrations	TT [ng/mL]	4.957	4.267	5.665
	FT [ng/mL]	0.081	0.060	0.101
	Bioavailable T [ng/dL]	1.660	1.260	2.320
	E2 [pg/mL]	24.96	21.3	28.7
	SHBG [nmol/L]	51.086	32.899	83.358
	DHEA [µg/mL]	0.522	0.252	1.126
Serum bone turnover marker concentrations	PINP [ng/mL]	977.667	751.000	1232.667
	CTX-I [ng/mL]	0.431	0.335	0.524
	PTH [pg/mL]	38.887	30.016	47.919
	Osteocalcin [ng/mL]	5.234	4.482	6.718
Serum concentrations [mg/L]	Ca	119.143	111.913	122.295
	K	164.035	155.194	181.081
	Zn	1.305	1.104	1.419
	Cu	0.971	0.771	1.093
	Fe	1.437	1.126	1.940
	Na	3292.449	3153.847	3376.195
	Cr	0.005	0.004	0.007
	P	169.532	151.525	189.662
	Mg	25.478	24.169	27.541
	Mn	0.007	0.006	0.008
Bone concentrations [mg/kg]	Ca	250,871.804	204,216.986	342,123.626
	K	762.146	628.072	1122.613
	Zn	207.630	182.095	235.525
	Cu	1.414	0.999	2.254
	Fe	90.163	52.325	168.250
	Na	9068.775	7100.015	12,180.974
	Cr	1.460	0.536	3.960
	P	143,891.177	136,803.311	176,665.264
	Mg	3306.299	2761.346	4351.710
	Mn	0.225	0.140	0.571
	Pb	2.926	2.092	4.178

Me—median; Q1—lower quartile; Q3—upper quartile; TT—total testosterone; FT—free testos-terone; bioavailable T—bioavailable testosterone; E2—estradiol; SHBG—sex hormone-binding globulin; DHEA—dehydroepiandrosterone; PINP—procollagen type I N-terminal propeptide; CTX-I—C-terminal telopeptide of type I collagen; PTH—parathyroid hormone; Ca—calcium; K—potassium; Zn—zinc; Cu—copper; Fe—iron; Na—sodium; Cr—chromium; P—phosphorus; Mg—magnesium; Mn—manganese; Pb—lead.

**Table 2:** Spearman’s rank correlation coefficients between local steroid receptor expressions in bone tissue, key accumulated elements, and serum parameters in the study group.

Parameter Pair	Spearman’s Coefficient (rs)	*p*-Value
ERα (bone) vs. PINP (serum)	+0.279	0.038
AR (bone) vs. CTX-I (serum)	+0.212	0.081
AR (bone) vs. Pb (bone)	+0.415	0.001
ERα (bone) vs. Mn (bone)	+0.312	0.022
ERα (bone) vs. BMI	−0.442	0.002

AR—androgen receptor; ERα—estrogen receptor alpha; Pb—lead; Mn—manganese; BMI—body mass index; PINP—procollagen type I N-terminal propeptide; CTX-I—C-terminal telopeptide of type I collagen—estrogen receptor alpha; Pb—lead; Mn—manganese; BMI—body mass index; PINP—procollagen type I N-terminal propeptide; CTX-I—C-terminal telopeptide of type I collagen; rs—Spearman’s rank correlation coefficient; *p*-value indicating statistical significance (*p* < 0.05).

**Table 3:** Immunohistochemical expression profiles of androgen receptors (AR) and estrogen receptors alpha (ERα) in the bone tissue of the study group.

Variable		*N* = 26		
		Me	Q1	Q3
Immunohistochemical reactions	percentage of ERα-positive cells	3.473	2.083	4.426
	percentage of AR-positive cells	10.140	7.656	13.310
Anthropometric parameters	Age [years]	66.000	64.000	71.000
	BMI [kg/m^2^]	29.225	27.118	30.992

Me—median; Q1—lower quartile; Q3—upper quartile; AR—androgen receptor; ERα—estrogen receptor alpha.

**Table 4:** Multivariate backward stepwise regression models identifying determinants of androgen receptor and estrogen receptor alpha expression in the bone tissue of men.

Variable	Variable	β	Standard Error	*p*
Final backward stepwise regression model for ARs	Age	0.53	0.26	0.055
	DHEA	−3.76	2.01	0.076
	E2	0.04	0.02	0.094
Final backward stepwise regression model for ERα receptors	BMI	−0.5	0.14	0.0018
	E2	0.03	0.01	0.0097
	SHBG	−0.03	0.01	0.0233
	DHEA	−1.74	0.79	0.0402

AR—androgen receptor; ERα—estrogen receptor alpha; DHEA—dehydroepiandrosterone; E2—estradiol; BMI—body mass index; SHBG—sex hormone-binding globulin; β—standardized regression coefficient; *p*—*p*-value.

**Table 5:** Multivariate backward stepwise regression analysis assessing the effect of steroid receptor expression in bone tissue on serum bone turnover markers in men, adjusted for age and body mass index.

Dependent Variable	Variable	β	*p*
PINP	Age	25.25	0.535
	BMI	−0.69	0.99
	ERα	142.87	0.0386
CTX	Age	−0.003	0.596
	BMI	0.013	0.122
	AR	0.007	0.0956
Osteocalcin	Age	0.09	0.546
	BMI	−0.22	0.275
PTH	Age	0.51	0.705
	BMI	0.32	0.856

AR—androgen receptor; ERα—estrogen receptor alpha; BMI—body mass index; DHEA—dehydroepiandrosterone; E2—estradiol; SHBG—sex hormone-binding globulin; β—standardized regression coefficient; *p*—*p*-value.

**Table 6:** Multivariate backward stepwise regression analysis assessing the effect of element concentrations in serum and bone tissue on steroid receptor expression, adjusted for age and body mass index.

	Receptor	Predictor	β	*p*
In serum	AR	P	0.08	0.0093
		Mg	−0.92	0.0283
		Fe	3.11	0.1287
	ERα	Cu	−4.29	0.1037
		BMI	−0.27	0.1178
In bone	AR	K	0.013	0.0001
		Pb	1.457	0.0009
		Age	0.760	0.0013
		BMI	−0.407	0.2004
	ERα	Mn	2.112	0.0409
		Age	0.138	0.2991

AR—androgen receptor; ERα—estrogen receptor alpha; P—phosphorus; Mg—magnesium; Fe—iron; Cu—copper; BMI—body mass index; K—potassium; Pb—lead; Mn—manganese; β—standardized regression coefficient; *p*—*p*-value.

## Data Availability

The data presented in this study are available on request from the corresponding author.

## Abbreviations

The following abbreviations are used in this manuscript:

AR	Androgen Receptor
ERα	Estrogen Receptor alpha
OA	Osteoarthritis
BMI	Body Mass Index
PINP	Procollagen I N-terminal Propeptide
CTX-I	C-terminal Telopeptide of Type I Collagen
Pb	Lead
Mn	Manganese
DHEAS	Dehydroepiandrosterone Sulfate
T	Testosterone
E2	Estradiol
SHBG	Sex Hormone-Binding Globulin
